# Using eHealth to Reach Black and Hispanic Men Who Have Sex With Men Regarding Treatment as Prevention and Preexposure Prophylaxis: Protocol for a Small Randomized Controlled Trial

**DOI:** 10.2196/11047

**Published:** 2018-07-16

**Authors:** Jacob J van den Berg, Taylor Silverman, M Isabel Fernandez, Kirk D Henny, Zaneta J Gaul, Madeline Y Sutton, Don Operario

**Affiliations:** ^1^ Center for Alcohol and Addiction Studies Department of Behavioral and Social Sciences Brown University Providence, RI United States; ^2^ College of Osteopathic Medicine Division of Health Professions Nova Southeastern University Fort Lauderdale, FL United States; ^3^ Centers for Disease Control and Prevention Atlanta, GA United States; ^4^ ICF Atlanta, GA United States

**Keywords:** pre-exposure prophlaxis, treatment as prevention, men who have sex with men, eHealth, blacks, African Americans, Hispanic Americans, Latinos, HIV, social media, sexual and gender minorities, telemedicine, acquired immunodeficiency syndrome

## Abstract

**Background:**

Black and Hispanic men who have sex with men in the United States continue to be disproportionately affected by HIV and AIDS. Uptake of and knowledge about biobehavioral HIV prevention approaches, such as treatment as prevention and preexposure prophylaxis, are especially low in these populations. eHealth campaigns and social media messaging about treatment as prevention and preexposure prophylaxis may help to fill this gap in knowledge and lead to increased uptake of such strategies; however, no evidence exists of the effects of these targeted forms of communication on treatment as prevention and preexposure prophylaxis uptake in these populations.

**Objective:**

We describe the protocol for a 3-part study aiming to develop and evaluate an eHealth intervention with information about treatment as prevention and preexposure prophylaxis for HIV-positive and HIV-negative black and Hispanic men who have sex with men.

**Methods:**

Phases 1 and 2 will involve focus groups and cognitive interviews with members of the target populations, which we will use to create a culturally tailored, interactive website and applicable social media messaging for these men. Phase 3 will be a small randomized controlled trial of the eHealth intervention, in which participants will receive guided social media messages plus the newly developed website (active arm) or the website alone (control arm), with assessments at baseline and 6 months.

**Results:**

Participant recruitment began in August 2017 and will end in August 2020.

**Conclusions:**

Public health interventions are greatly needed to increase knowledge about and uptake of biobehavioral HIV prevention strategies such as treatment as prevention and preexposure prophylaxis among black and Hispanic men who have sex with men. eHealth communication campaigns offer a strategy for engaging these populations in health communication about biobehavioral HIV prevention.

**Trial Registration:**

ClinicalTrials.gov NCT03404531; https://www.clinicaltrials.gov/ct2/show/NCT03404531 (Archived by WebCite at http://www.webcitation.org/70myofp0R).

**Registered Report Identifier:**

RR1-10.2196/11047

## Introduction

### Background

There were an estimated 973,846 people living with HIV in the United States as of 2015; the US Centers for Disease Control and Prevention (CDC) further estimated that roughly 38,500 new infections occur annually [1]. Men who have sex with men (MSM) accounted for about 70% of those new infections in 2015, despite constituting just 2% of the country’s population [2]. Moreover, roughly 70% of the new diagnoses that year were in black and Hispanic MSM [2].

Black and Hispanic MSM continue to be disproportionately affected by HIV/AIDS in the United States. These racial and ethnic disparities in HIV/AIDS rates are also apparent in the Providence, Rhode Island Metropolitan Area, where new HIV/AIDS diagnoses among MSM increased between 2007 and 2011 even as total new diagnoses declined [3]. Moreover, 30% of individuals with a new diagnosis in Rhode Island from 2009 to 2013 also presented with AIDS, with MSM status being the leading risk factor, suggesting that a significant number of MSM in Rhode Island are only reaching care late in the course of their disease [3].

Effective biobehavioral interventions for HIV prevention in such high-risk populations include treatment as prevention (TasP) and preexposure prophylaxis (PrEP). TasP refers to the use of antiretroviral treatment to decrease the risk of HIV transmission between serodiscordant partners by reducing the viral load in the infected individual’s fluids to very low levels. TasP has been shown to have the potential to reduce HIV transmission to the partner without HIV by more than 96% [4]. PrEP is the daily ingestion of an oral single-tablet combination antiretroviral treatment by HIV-uninfected individuals. PrEP has been shown to have a greater than 90% chance of preventing HIV acquisition in adherent individuals, as confirmed by multiple studies [5-9]. However, knowledge about and uptake of PrEP and TasP remain low among black and Hispanic MSM [10,11]. Though results vary across studies, PrEP uptake is estimated at just 9.8% for black MSM and 6.6% for Hispanic MSM [12]. Barriers to PrEP uptake identified by previous research include lack of cultural competency in public health initiatives, stigma related to homosexuality and HIV serostatus, lack of targeted internet outreach, and low health literacy in the target populations [13].

One highly promising strategy to increase awareness, knowledge, behavioral intentions, and potential uptake of public health interventions that is underused in this context is eHealth, which refers to the use of a range of electronic technologies (eg, online social networking sites and apps, YouTube, and interactive websites) to provide health information [14-17]. eHealth has recently moved to the forefront of health communication because of its cost effectiveness, high levels of accessibility and acceptability in many populations, and effectiveness in previous public health campaigns [16,18-22]. Of all American adults, 84% use the internet [23] and MSM of all racial and ethnic groups have been found to have high rates of participation on online social media sites [24]. Recent research found that MSM frequently prefer information about HIV prevention (eg, PrEP) to be disseminated in electronic forms such as email and websites [21]. However, our recent review of the published literature did not find that any evaluations of health interventions using social media increased TasP or PrEP uptake in these high-risk populations, demonstrating its novelty.

Further, cultural tailoring, or the use of targeted messages for certain populations on the basis of known subgroup differences, has also been found to increase efficacy compared with generic health messaging [25,26]. For example, Kreuter and colleagues used messages to prompt African American women to increase their rates of mammography and healthy food consumption by tailoring those behavioral changes toward values such as religiosity, collectivism, and racial pride [26]. Yet few existing websites or online sources of HIV prevention information are tailored to black and Hispanic MSM. In addition, data on eHealth, and particularly social media’s utility for minority populations, remain limited.

### Objective

We describe the protocol of a 3-part study aiming to develop and evaluate an eHealth intervention with information about TasP and PrEP for HIV-positive and HIV-negative black and Hispanic MSM. One part of this project will involve the creation of a culturally tailored, interactive website for these men in the Providence Metropolitan Area and of similarly tailored social media messaging to promote the website and prompt participants to access it. The population-specific content and all aspects of the website and messages will be based heavily on data gathered from members of the population in the initial 2 phases of the study. The messages will also be grounded in the information-motivation-behavioral skills (IMB) and social cognitive theory (SCT) frameworks, which are widely accepted evidence-based approaches for promoting behavioral changes related to HIV risk reduction [27-32]. We hypothesize that using theory-based and culturally relevant social media messages will increase knowledge of TasP and PrEP among black and Hispanic MSM, positively affect their attitudes and behavioral intentions toward the interventions, and ultimately increase uptake. The overall objective of this study is to improve HIV prevention strategies among high-risk minority MSM through novel use of eHealth.

## Methods

### Study Design

This will be a 3-part study that includes focus groups and cognitive interviews to develop an eHealth intervention for HIV-positive and HIV-negative black and Hispanic MSM, and a small randomized controlled trial (RCT) to evaluate that intervention. In phase 1, we will use 4 to 6 focus groups of 5 to 8 participants each to learn about TasP, PrEP, and social media use in the target population, as well as obtaining feedback on an existing interactive website (Men2MenRI [33]). Members of our team previously developed the website targeting white MSM in Rhode Island. Phase 2 will use cognitive interviews with 8 participants and an open pilot with 16 participants to develop and assess the acceptability of IMB- and SCT-grounded social media message content designed to motivate and encourage access to our newly developed website. In phase 3, we will conduct a small RCT (n=100 participants, with 50 in each arm) comparing the combination of sending social media messages plus the website (active arm) versus the website alone (control arm). Study materials (eg, recruitment flyers, interview guides, questionnaires, website, and social media messages) and findings (eg, qualitative and quantitative data) will also be assessed by a community advisory board made up of 6 to 12 members from the local community who reflect the target groups. The study is approved by the Brown University Institutional Review Board (#1612001661), Providence, Rhode Island, USA, and the US National Center for HIV/AIDS, Viral Hepatitis, STD, and TB Prevention’s project determination process. This study is also registered on ClinicalTrials.gov (NCT03404531).

### Participant Recruitment

Participants will be HIV-positive and HIV-negative black and Hispanic MSM who are 18 years of age and older living or working in the Providence Metropolitan Area (all of Rhode Island and Bristol County, Massachusetts). Participants must also fit the CDC definition of high risk—that is, have engaged in condomless anal intercourse within the past 6 months; be biologically male and identify as male; and be able to give written informed consent in English or Spanish. For phase 3, participants must also report not taking PrEP if they are HIV-negative, or in HIV care or taking treatment if they are HIV-positive, at the time of enrollment in the study. We will recruit participants for all phases of the study using a variety of methods, including advertisements online and on public transportation; posted signs and flyers in local community and commercial venues; and in-person outreach at places where the target population congregates, such as community-based organizations, clinics, bars, and clubs. Individuals interested in participating will be screened for eligibility over the phone using the aforementioned criteria. Participants will provide written informed consent prior to participating in the focus group, cognitive interview, or baseline assessment.

### Procedures and Interventions

For all phases, participants will complete a demographics form at the time of their enrollment. For phase 1, we will attempt to stratify the groups by race/ethnicity (black vs Hispanic) and HIV serostatus (HIV-positive vs HIV-negative). Focus groups will be facilitated by 2 trained staff members in English or Spanish using a semistructured discussion guide and will last roughly 2 hours. In both parts of phase 2, we will attempt to have a balance of participants from each of the 4 categories from phase 1 (black HIV-negative, Hispanic HIV-negative, black HIV-positive, and Hispanic HIV-positive). The cognitive interviews will be 2- to 3-hour sessions in English or Spanish using a semistructured interview guide with open-ended questions, also administered by a trained member of the research team. All phase 1 focus groups and phase 2 interviews will be digitally recorded and professionally transcribed. For the open pilot portion of phase 2, participants will attend an individual information session at the study location, where they will be provided with a broad description of the intervention and view the website. They will then participate in the newly developed intervention (website plus messages) for 1 week. Lastly, participants will return to the study location to complete a brief (1-hour) individual in-depth interview about the website and messages. Based on the feedback we receive during these interviews, we will alter intervention materials and procedures prior to the implementation of the study as a small RCT during phase 3.

We will randomly assign phase 3 participants to have access to either the website with social media messages (intervention condition) or the website alone (control condition). Participants will be stratified by race/ethnicity and HIV serostatus as in phases 1 and 2. We will use block random assignment in batches of 10, by HIV status and race/ethnicity, to keep the sizes of the intervention or comparison groups similar. Participants will physically come to the study site at enrollment for study description, consent procedures, website demonstration, randomization, demographic and HIV treatment and prevention history information collection, and baseline assessment; then they will come again 6 months later for the follow-up assessment. Participants will be assigned a research identification number, which will be used for all data collected to ensure confidentiality. Participants will be compensated in cash (all in US $) $40 for the focus group, $50 for the cognitive interview, $35 for the open pilot, and $80 for the small RCT ($40 for the baseline assessment and $40 for the 6-month follow-up) plus a potential $10 for transportation or child care costs incurred to attend study sessions. Eligible individuals will be allowed to participate in only 1 phase of the study.

Each phase of the study will provide unique information that will then inform the following phases. We will use focus group data from phase 1, which will include gathering feedback on the existing Men2MenRI website and to learn about TasP, PrEP, and social media use in the target populations, to create our new website and social media messages in a way that is audience specific. We will then use cognitive interviewing in phase 2 to further refine and develop the website and messages to be tailored to participant serostatus and race/ethnicity. Lastly, we will conduct an open pilot to evaluate and improve study procedures prior to the implementation of the small RCT in phase 3.

### Measures

Phases 1 and 2 measures will involve using qualitative interview guides, informed by the extant literature and developed by the team, to identify important themes in the data. For phase 3, participants will complete the study assessment at enrollment (baseline) and at a 6-month follow-up, which will be digitally administered using iPads. The assessment will include self-report questions about HIV testing history, date(s) of previous testing, source(s) of previous testing, viral load, CD4 count, linkage and retention in care, TasP uptake, HIV medication, adherence and viral load if HIV-positive, and consideration of PrEP, including barriers and facilitators and its uptake, if HIV-negative. The assessment will also include measures that we will develop from the focus group data to assess participants’ knowledge, attitudes, and behavioral intentions toward TasP and PrEP use. Validated measures will include event-level characteristics of sexual risk episodes over the past 3 months, using the timeline follow-back interview method, with information on sexual activities, relationship with partner(s), partner gender, use of alcohol or drugs prior to or during, and use of condoms for each episode. Phase 3 will also involve fidelity monitoring of participants’ use of the intervention, including information about when they check or receive messages, whether they reply to messages or input information via messages, and whether they log calls to study staff.

### Study Outcomes

The primary outcome of the study will be levels of and increases in TasP or PrEP uptake in the intervention group compared with the control group, as determined by using assessment data from phase 3. Secondary outcomes include increases in levels of TasP- and PrEP-specific knowledge, favorable attitudes, and behavioral intentions regarding TasP and PrEP among participants in the intervention group. Lastly, we will explore decreases in sexual risk behaviors in the intervention group relative to the control.

### Planned Analyses

For the qualitative analyses in phases 1 and 2, we will independently read and code professional transcriptions of the focus groups and interviews, and will convene regularly to discuss emerging themes and systematically establish categories. Finalized thematic codes will provide an exhaustive categorization tool of concepts and themes described by participants. Multiple coders will analyze subsets of data, interrater reliability will be assessed, and any discrepancies will be resolved with discussion.

In phase 3, we will first assess the randomization process by comparing baseline demographic variables of participants in each arm; we will incorporate any variable for which randomization did not result in equal proportions in each arm into the multivariate models as a potential confounding variable. Next, we will conduct primary inferential analyses to test our primary hypothesis (ie, that messages sent over social media will increase TasP and PrEP uptake among the men in the intervention arm versus the control arm) followed by our secondary (increases in knowledge of, more favorable attitudes toward, and behavioral intentions regarding TasP and PrEP use between men in the intervention and control arms) and exploratory (decrease in sexual risk behaviors among men in the intervention arm in comparison with the control arm) hypotheses. Since the primary and tertiary outcome measures are binary, we will first compare proportions by randomization arm separately for each measurement period using standard bivariate analytic techniques (eg, Spearman rank-order correlations, odds ratios, and Fisher exact test). We will use ordinary least squares regression on our secondary measures of knowledge, attitudes, and behavioral intentions, which we will assess using continuous scales. We will contrast outcomes in the intervention versus comparison groups over time via generalized linear mixed models. Given the binary nature of our TasP and PrEP uptake and sexual risk measures, we will specify a logit link function for our generalized linear mixed models.

We will follow an intent-to-treat design and will include data from all enrolled participants in the analysis, regardless of level of intervention use. We will compare characteristics of participants who are lost to follow-up with those who are evaluated to assess for systematic patterns that could influence results. Following intent-to-treat principles, we will include all randomly assigned participants in the data analyses.

Effect-size estimates determined through the small RCT will be essential in the design of a larger and fully powered efficacy study that tests intervention effects on TasP and PrEP uptake and behavioral risk reduction. We are well aware of the limitations of relying exclusively on small-scale pilots to determine whether novel intervention approaches are promising, namely that sizable standard errors are associated with effect sizes due to the small sample size. However, we are primarily interested in exploring the pattern of results for any evidence of support for the intervention's influence on the primary and secondary outcomes. Our proposed sample size of 100 will be sufficient to detect an effect size of 0.3 or greater between the 2 groups, with 80% power and a 2-tailed alpha level of .05. Furthermore, we will be able to detect similar effect sizes (of ≥0.3) with respect to condomless anal intercourse and secondary analysis of social-cognitive behaviors.

## Results

Development of this project began in May 2016. Participant recruitment for phase 1 began in August 2017 and is scheduled to be completed in August 2018. Phase 2 recruitment will begin in August 2018 and is scheduled to be completed in August 2019. Phase 3 recruitment will begin in August 2019 and is scheduled to be completed in August 2020.

## Discussion

### Principal Findings

The goal of this project is to add to the science about HIV prevention interventions and eHealth among black and Hispanic MSM in the United States. Successful interventions are urgently needed to reduce the burden of HIV for racial and sexual minority men. If the intervention is effective, social media messaging and culturally tailored online information could be a low-cost, high-impact way to increase uptake of HIV prevention methods in these high-risk populations. This study will also generate much-needed data on social media’s utility for eHealth interventions among HIV-positive and HIV-negative black and Hispanic MSM. While eHealth has substantial potential, it remains hard to assess its ability to motivate behavioral change given the lack of data available, particularly for racial and ethnic minority MSM.

### Study Limitations

There are study limitations that are important to highlight. First, given that this study will focus on HIV-positive and HIV-negative black and Hispanic MSM living in the Providence Metropolitan Area, our findings may not generalize to similar men living in other areas of the United States. Second, our results will be based on data that are self-reported by participants, which is subject to recall and social desirability biases. Third, the website and social media messages that we will develop, based on feedback from the focus groups and cognitive interviews, will be limited by the constant evolution of technology and its outpacing of research.

### Study Strengths

Despite these limitations, this study has several strengths. First, it uses a combined approach to TasP and PrEP uptake that leverages advances in social media as a platform for motivating behavioral change to potentially overcome some of the noted barriers to use of HIV treatment and prevention services. Second is the use of social media messages grounded in IMB and SCT to provide targeted groups with timely information and motivational cues to access the website about TasP and PrEP. A third advantage is that the website will be culturally tailored and specifically designed by and for HIV-positive and HIV-negative black and Hispanic MSM. Fourth, findings from this research have the potential to influence policy guidelines and recommendations for TasP and PrEP uptake for high-risk groups.

### Conclusions

Development and evaluation of this newly developed website will allow for improved implementation and delivery of TasP and PrEP, and will help prevent HIV acquisition and transmission among high-risk HIV-positive and HIV-negative black and Hispanic MSM in the United States. We will use the results of this study for a larger-scale trial to test the effectiveness of the combined messages, plus the website, on behaviors leading to HIV treatment and prevention for these 2 populations. In addition, we will conduct future research to examine the long-term effects of the website on TasP and PrEP adherence among their users.

## Abbreviations

CDC: Centers for Disease Control and Prevention
IMB: information-motivation-behavioral skills
MSM: men who have sex with men
PrEP: preexposure prophylaxis
RCT: randomized controlled trial
SCT: social cognitive theory
TasP: treatment as prevention
